# Relieving Effect of *Artemisia ordosica Krasch* Extract on DSS-Induced Colitis by Regulating Immunity, Antioxidant Function, Gut Microbiota, and Bile Acid Metabolism in Mice

**DOI:** 10.3390/antiox14010045

**Published:** 2025-01-02

**Authors:** Min Jiang, Xuekai Zhang, Xiao Jin, Binlin Shi, Yuanqing Xu, Zheqi Wang

**Affiliations:** 1 College of Animal Science, Inner Mongolia Agricultural University, Hohhot 010018, China; jiangmin080808@163.com (M.J.); zxk1101101102023@163.com (X.Z.); yaojinxiao@aliyun.com (X.J.); shibinlin@yeah.net (B.S.); 2 Institute of Grassland Research of CAAS, Hohhot 010010, China

**Keywords:** *Artemisia ordosica Krasch* extract, colitis, immune response, antioxidant function, gut microbiota, bile acid metabolism

## Abstract

*Artemisia ordosica Krasch*, a traditional Chinese herbal medicine, possesses antibacterial, antiviral, and anti-inflammatory properties. The aim of this experiment was to investigate the therapeutic effect of *Artemisia ordosica Krasch* extraction (AOE) in treating colitis induced by dextran sulfate sodium (DSS) in mice. The in vitro antioxidant activity of AOE was evaluated by assessing its iron reduction capacity and scavenging capacity towards 1,1-diphenyl-2-picrylhydrazyl (DPPH) and hydroxyl radicals (·OH). The protective effect of AOE on colitis in mice was determined by monitoring key indicators such as body weight, colon length, and survival rate in mice, as well as by assessing the expression of colon-related genes and cytokine levels. We evaluated the impact of AOE on intestinal microbiota by measuring the 16s sequencing of cecal contents and bile acid metabolism. The results showed that the iron reduction capacity of AOE was positively correlated with its concentration. The half-maximal inhibitory concentrations (IC_50_) for scavenging DPPH and hydroxyl radicals were 3.126 mg/mL and 6.139 mg/mL, with a 95% confidence interval of 95%. In vivo studies demonstrated that AOE reduced DSS-induced colitis in mice by increasing the colon length, enhancing antioxidant enzyme activity, inhibiting inflammatory cell infiltration, suppressing the formation of TNF-α and IL-6, and reducing malondialdehyde (MDA) levels. qPCR analysis revealed that AOE reversed the down-regulation of *Claudin* mRNA expression, and altered the composition of cecal microbiota, thus mitigating DSS-induced colitis. AOE plays a crucial role in alleviating colitis in mice and effectively improves DSS-induced colitis, highlighting its potential as a therapeutic agent for inflammatory bowel diseases.

## 1. Introduction

Ulcerative colitis (UC) is a non-specific chronic inflammatory disease that primarily targets the colon. Typical clinical manifestations of UC encompass abdominal discomfort, frequent diarrhea, rectal bleeding, and unintended weight reduction [1,2]. The etiology of UC is multifaceted, encompassing genetic predisposition, disruptions in the epithelial barrier, alterations in the intestinal microbiome, immune system dysfunctions, and environmental triggers [2]. At present, anti-inflammatory treatment is the main method to treat colitis; the amino acid salicylate, corticosteroids, and immunosuppressants are frequently prescribed in clinical settings as anti-inflammatory medications for the management of colitis [3,4]. However, these drugs only show good effects in some patients, and they are also associated with serious side effects such as infection and cancer risk [5].

The intestinal mucosal barrier, in conjunction with the intestinal microbiota, is an indispensable factor for maintaining intestinal health [5]. The typical characteristics of intestinal microecological imbalance are a decrease in intestinal flora diversity, imbalance of bacterial phyla, increase in the count of harmful metabolites, and decrease in the count of beneficial metabolites [6]. The intricately interconnected epithelial cells that compose the intestinal mucosal barrier serve as a vital line of defense, safeguarding against the invasion of pathogenic bacteria and harmful substances [7]. In the context of UC, the disruption of the intestinal barrier is accompanied by alterations in the levels of tight-junction-linked proteins [8]. The disruption of tight-junction-related proteins allows pathogenic bacteria to enter the lamina propria from the lumen, triggering inflammatory responses that exacerbate intestinal inflammation and epithelial damage [9]. Furthermore, gastrointestinal homeostasis is jointly regulated by bile acids (BAs) and intestinal microbiota. The intestinal microbiota exerts a modulating influence on the synthesis, metabolism, and reabsorption of BAs, and conversely, the synthesis and metabolism of BAs regulate the diversity and abundance of the intestinal microbiota, creating a bidirectional interplay [10]. BAs, the primary constituents of bile, exhibit dual roles: they possess potent antibacterial properties and serve as signaling molecules, engaging with the takeda G-protein-coupled receptor 5 (TGR5) and the nuclear receptor farnesoid X receptor (FXR), thereby mediating various biological responses [11]. They play a crucial role in lipid, glucose, and energy metabolism, thereby contributing significantly to the preservation and maintenance of the intricate balance and stability of the intestinal microbial homeostasis [12].

*Artemisia* species occupy a prominent position among the most sought-after plants in Chinese traditional herbal medicine, being routinely employed in the therapeutic management of diverse ailments encompassing malaria, hepatitis, cancer, and inflammatory conditions, as well as infections stemming from fungi, bacteria, and viruses [13]. Due to their abundant nutritive content and bioactive compounds, the *Artemisia* genus of plants and their derivatives can serve as natural feed additives in Chinese herbal medicine, significantly contributing to enhancing animal growth performance, upgrading animal product quality, and bolstering the immunocompetence and antioxidant action capabilities of animals [14,15,16,17]. *Artemisia ordosica Krasch*, belonging to the genus *Artemisia* within the Asteraceae family, is widely distributed in the northwest of China and the western part of Inner Mongolia [18]. *Artemisia ordosica Krasch* extraction (AOE) is obtained by using *Artemisia ordosica Krasch* as the raw material. After processing, it is dissolved in water as the solvent and then concentrated to obtain an extract that contains active ingredients and maintains its structural integrity. Our team’s previous studies have shown that AOE is a natural substance that can improve animals’ immune and antioxidant functions while enhancing their growth performance [19,20]. It has been confirmed that active ingredients such as flavonoids and polysaccharides extracted from *Artemisia ordosica Krasch* possess anti-inflammatory, antioxidant, immunomodulatory, and growth-promoting effects [21,22,23]. Moreover, clinical studies have successfully employed decoctions of herba *artemisiae scopariae*, a plant with characteristics akin to *Artemisia ordosica Krasch*, achieving satisfactory outcomes [24]. In recent years, there has been a decline in the number of reports investigating the potential of AOE to mitigate the symptoms of colitis induced by DSS in mouse models. Given the immense potential of *Artemisia ordosica Krasch*, we hypothesize that its decoction-prepared and freeze-dried extract may help reduce the severity of ulcerative colitis. Therefore, the objective of this study was to investigate the impact of AOE on DSS-induced colitis in mice with the aim of contributing to the advancement of alternative treatment methods for ulcerative colitis.

## 2. Materials and Methods

### 2.1. Reagents and Equipment

Ascorbic acid, hydrogen peroxide, phosphate buffer, 1,1-diphenyl-2-picryl-hydrazyl radical (DPPH), anhydrous ethanol, potassium ferricyanide, trichloroacetic acid, ferric chloride, ferrous sulfate, and salicylic acid were procured. Dextran sulfate sodium (DSS) was purchased from MeilunBio (Dalian, China) and the test kit including tumor necrosis factor-α (TNF-α) and interleukin-6 (IL-6) was purchased from Wuhan Colorful Gene Biological Technology Co., Ltd. (Wuhan, China). Antioxidant kits including total antioxidant capacity (T-AOC), glutathione peroxidase (GPx), total superoxide dismutase (T-SOD), catalase (CAT), and malondialdehyde (MDA) were purchased from Nanjing Jiancheng Bioengineering Institute (Nanjing, China), and TRIzol reagents were purchased from Bio-Rad Company (Shanghai, China). SHZ-88-1 water bath constant temperature shaker, RE-5298 rotary evaporation device (Shanghai Yarong Biochemical Instrument Equipment Co., Ltd., Shanghai, China), SHB-3 circulating water multi-purpose vacuum pump (Zhengzhou Great Wall Technology & Trade Co., Ltd., Zhengzhou, China), ALPHA 1-2LD plus vacuum freeze dryer (Marin Christ Company, Osterode am Harz, Germany), TD4Z electric centrifuge (Hunan Kaida Scientific Instrument Company, Changsha, China), and V-1000 visible spectrophotometer (Shanghai Ao Yi Instrument Co., Ltd., Shanghai, China) were also procured.

### 2.2. Preparation of AOE

The plant name *Artemisia ordosica Krasch* was checked with The Plant List (http://www.theplantlist.org) and The World Flora Online (http://www.worldfloraonline.org) accessed on 26 April 2024. The sample of *Artemisia ordosica Krasch* used in this study was collected from Ordos City, Inner Mongolia, China. We obtained the aqueous extract of AOE utilizing the optimal extraction method that we had previously identified and refined through our research efforts as follows. The entire plant of *Artemisia ordosica Krasch* was ground into powder. Liquid–solid ratio of 35:1 mL/g and a water bath temperature of 70 °C were maintained for 10 h. The extracts were mixed and introduced into a rotary evaporator and concentrated into a paste at 70 °C under vacuum. This paste was then dried further to yield a medicinal powder sample comprising 23.4% of the original extract.

### 2.3. Analysis of AOE Active Ingredients

To prepare the biological samples for UPLC-MS/MS analysis, vacuum freeze-drying technology was applied by placing them in a Scientz-100F lyophilizer (Scientz, Ningbo, China). Subsequently, the dried samples were finely ground into a powder using an MM 400 Retsch grinder (Retsch, Haan, Germany, 30 Hz, 1.5 min). A precise 50 mg aliquot of this powdered sample was then weighed and mixed in with 1200 μL of a pre-cooled (−20 °C) 70% methanolic aqueous–methanolic mixture featuring an internal standard extract. The compound was vortexed for 30 s every 30 min, totaling six vortexing intervals. After centrifugation (rotation speed 12,000 rpm, 3 min), the supernatant was carefully aspirated and the sample was passed through a 0.22 μm microporous membrane filter to remove any particulates before undergoing UPLC-MS/MS analysis.

### 2.4. Determination of AOE Antioxidant Activity

(1) The DPPH free radical scavenging rate was slightly modified by referring to the method of Baliyan [25]. We accurately absorbed 1 mL samples with concentrations of 0.5, 2, 4, 6, 8, and 10 mg/mL in test tubes, added 1 mL DPPH–ethanol solution (0.2 mmol/L), mixed, allowed reaction in darkness at room temperature for 30 min, centrifuged at 5000 r/min for 10 min, adjusted to zero with anhydrous ethanol, and determined the absorbance at 517 nm. Vitamin c (Vc) solution was used as the control.

DPPH free radical scavenging rate (%) = [1 − (A_0_ − A_1_)/A_2_] × 100

A_0_ is the absorbance of 1 mL aqueous extract solution + 1 mL DPPH solution.

A_1_ is the absorbance of 1 mL aqueous extract solution + 1 mL anhydrous ethanol solution.

A_2_ is the absorbance of 1 mL anhydrous ethanol solution + 1 mL DPPH solution.

(2) Hydroxyl radical scavenging ability was slightly modified according to Barreto’s method [26]: samples of 1 mL of AOE sample solution with concentrations of 0.5, 2, 4, 6, 8, and 10 mg/mL were accurately pipetted into each tube. Then, 1 mL of FeSO_4_ (9 mmol/L) and 1 mL of salicylic acid–ethanol solution (9 mmol/L) were added, mixed thoroughly, and allowed to stand for 10 min. Subsequently, 1 mL of H_2_O_2_ (8.8 mmol/L) was added and the reaction was carried out at 37 °C for 30 min. Finally, the absorbance was measured at 510 nm.

OH radical scavenging rate (%) = [1 − (A_2_ − A_1_)/A_0_] × 100

A_1_ is the absorbance of the solution without salicylic acid and ethanol.

A_2_ is the absorbance of samples of different concentrations.

A_0_ is the absorbance of the blank group (with sample solution replaced with distilled water).

(3) Iron reducing ability: referring to Arika’s method [27], 1 mL of AOE sample solution in concentrations of 0.5, 2, 4, 6, 8, and 10 mg/mL was accurately pipetted, and 1 mL phosphate buffer (0.2 mmol/L) with a pH of 6.6 and 2 mL of 1% potassium ferricyanide (1.0 g/100 mL) were added. The compound was then homogenized and incubated in a water bath at 50 °C for 20 min before being cooled. Subsequently, 1 mL of 10% trichloroacetic acid was added, and the compound was homogenized and centrifuged at 3500 rpm for 10 min. A quantity of 2.5 mL of the supernatant was then taken, and 2.5 mL of distilled water and 0.3 mL of 0.1% ferric chloride were added. After homogenization and standing for 10 min, the absorbance was measured at 700 nm.

Reducing capacity (A700) = (A_1_ − A_0_)

A_1_ is the absorbance of the sample solution with different concentrations.

A_0_ is the absorbance of distilled water instead of polysaccharide solution.

### 2.5. Treatment of Experimental Animals

The procedures for performing experiments that involved animals were granted approval by the Animal Research and Ethics Committee of the College of Animal Science at Inner Mongolia Agricultural University, Hohhot, China. Thirty specific pathogen-free (SPF) males were housed in a temperature-regulated environment that adhered to a strict 12 h light/dark cycle at 22~25 °C. These mice were allowed access to rodent chow and tap water ad libitum. Before the formal experiment, mice were acclimated in animal facility for 6 days. Mice were randomly segregated into 3 groups with 10 in each group based on body weight, including CON, 3% DSS, and 3% DSS + 1% AOE groups.

DSS and AOE were supplemented in autoclave water. All animal experiments were performed in accordance with the National Standard Guidelines for Ethical Review of Animal Welfare (GB/T 35892-2018) [China National Standardization Technical Committee for Laboratory Animals]. Each mouse’s weight was measured daily throughout the trial. The evaluation of disease activity index (DAI) was conducted by combining weight loss, diarrhea, and blood in the stool to gauge the severity level of colitis (Table A1) [28,29].

### 2.6. Sample Collection and Processing

On day 19 of the formal experiment, the mice were anesthetized with ether, and their eyeball blood was promptly collected. The blood samples underwent centrifugation at 3500× *g* for 10 min, and the supernatant was carefully aspirated and stored at −80 °C for subsequent analysis. Subsequently, the abdominal cavity was surgically opened, and the spleen, heart, liver, and kidneys were rapidly excised and accurately weighed. The entire colon and cecum were excised, measured, and photographed for documentation. The cecal contents and colonic tissue were preserved at −80 °C for further examination. A 1 cm segment of colon was excised, rinsed with phosphate buffer saline (PBS), and immersed in 4% paraformaldehyde fixative for histological slicing and staining. The leftover colon tissue was preserved at −80 °C for future analysis.

### 2.7. Histological Analysis

Colon tissue sections were fixed with 4% paraformaldehyde, dewaxed with xylene and high-concentration alcohol, and finally stained with hematoxylin and eosin.

### 2.8. Determination of Inflammatory Factors and Antioxidant Markers in Colon and Serum

The colon tissue was made into a 10% homogenate with physiological saline. The concentrations of TNF-α and IL-6 in colon tissue and serum were determined according to the instructions provided by ELISA kit (Wuhan Colorful Gene Technology Co., Ltd., Wuhan, China). The contents of CAT, T-SOD, MDA, T-AOC, and GPx in colon were determined according to commercial reagents and provided detection procedures.

### 2.9. Real-Time Quantitative PCR

Using TRIzol reagents, RNA was isolated from the samples of colon tissue, and cDNA was produced by means of reverse transcription kits. qPCR was performed using LightCycler96 PCR apparatus and the MagicSYBR Mixtures kit. The qPCR protocol involves the following steps: pre-denaturation at 95 °C for 30 s; PCR amplification stage with 95 °C for 5 s followed by 60 °C for 34 s; and melting curve analysis with 95 °C for 15 s, 60 °C for 1 min, and finally 95 °C for 15 s. The number of cycles ranges from 35 to 45. The relative alteration in gene expression was performed by 2^−ΔΔCt^. The primer sequences are provided in Table A2.

### 2.10. Analysis of Intestinal Microbial Community Based on 16sRNA

The CTAB was employed to extract the genomic DNA present within the cecal contents and agarose gel electrophoresis was utilized to determine both the purity and the concentration of the extracted DNA. An appropriate quantity of sample DNA was taken and placed into a centrifuge tube, where it was then diluted with sterile water to a concentration of 1 ng/μL. We used New England Biolabs’ Phusion High-Fidelity PCR Master Mix with GC Buffer using the diluted genomic DNA as template and specific primers with Barcode according to the selection of sequencing region. We utilized a high-efficiency high-fidelity enzyme for PCR, thereby guaranteeing both efficiency and precision in the amplification process.

### 2.11. Metabolomics Analysis

Cecal content samples (20 mg) were extracted with 500 μL methanol after being ground using a ball mill, then 10 μL aliquot of the internal standard mixed solution (1 μg/mL) was added into the extract to serve as a reference point for quantification purposes. To precipitate the proteins, the samples were placed at −20 °C for a period of 10 min. Subsequently they were centrifuged for 10 min (12,000× *g*, 4 °C). After centrifugation, supernatant was passed through protein precipitation plate for further LCMS analysis.

### 2.12. Statistical Analysis

The concentration of extract was taken as the horizontal coordinate and the inhibition rate as the vertical coordinate. The curve was fitted using GraphPad Prism 8.0.2 to obtain the IC_50_ value. Preliminary analysis and organization of experimental data were conducted using Excel 2021 and one-way analysis of variance (ANOVA) was performed using SAS 9.2. The data are shown as means ± SDs. Six animals in separate group were used for analyzing the biochemical indicators of the serum and colon. A *p*-value of less than 0.05 was taken as the threshold for determining statistical significance. Note: * *p* < 0.05, ** *p* < 0.01, and *** *p* < 0.001.

## 3. Results

### 3.1. AOE Active Ingredients

**Figure 1:**
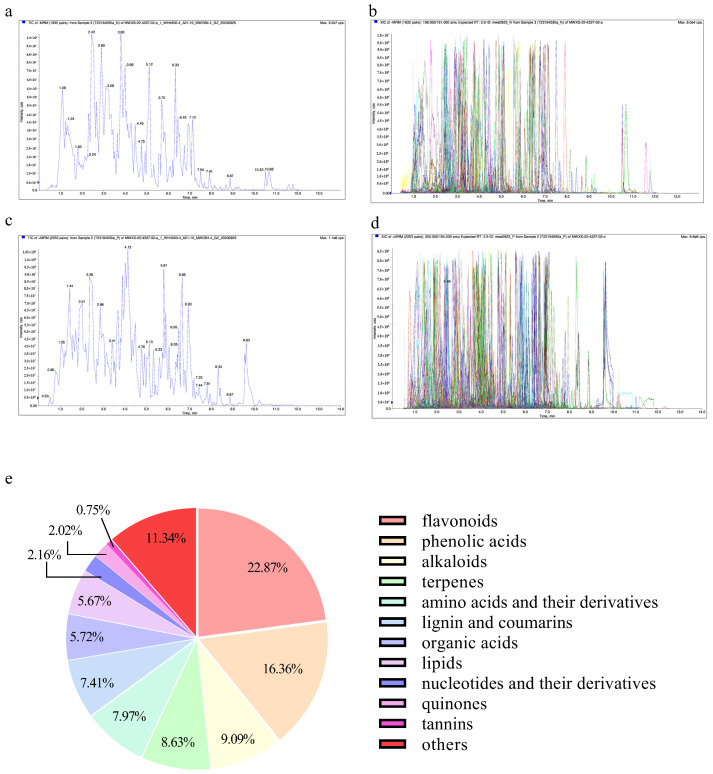
Analysis of AOE active ingredients. (**a**) Total ion current of mass spectrometry analysis for mixed sample quality (e+). (**b**) MRM detection of multimodal maps (e+). (**c**) Total ion current of mass spectrometry analysis for mixed sample quality (e−). (**d**) MRM detection of multimodal maps (e+). (**e**) Pie chart of major substances. Note: in Figures (**b**,**d**), each chromatogram peak of a different color represents a detected metabolite.

Through the scan + MRM mode of liquid chromatography–tandem mass spectrometry (LC-MS/MS) for the qualitative analysis of AOE active ingredients, we identified 2133 substances, including flavonoids, phenolic acids, alkaloids, terpenes, amino acids and their derivatives, lignin and coumarin, organic acids, lipids, nucleotides and their derivatives, quinones, tannins, and others. These substances accounted for 22.87%, 16.36%, 9.09%, 8.63%, 7.97%, 7.41%, 5.72%, 5.67%, 2.16%, 2.02%, 0.75%, and 11.34% of the main components, respectively (Figure 1).

### 3.2. Determination of Antioxidant Activity of AOE

#### 3.2.1. DPPH Free Radical Scavenging Ability of AOE

**Figure 2:**
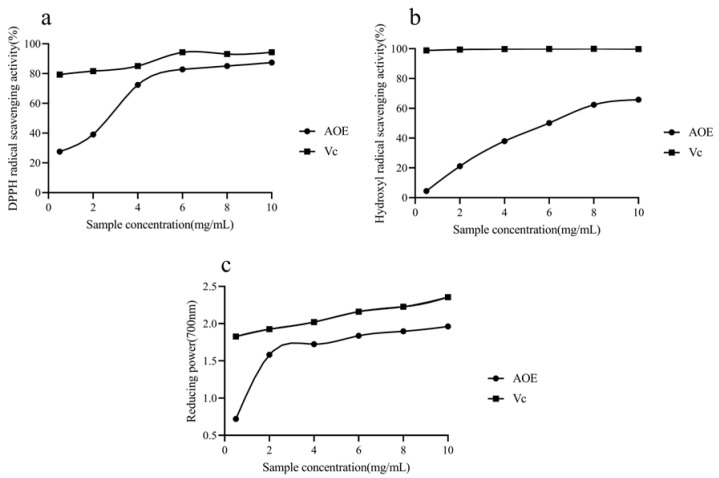
Antioxidant capacity of AOE in vitro. (**a**) Determination results of scavenging DPPH free radical. (**b**) Hydroxyl free radical. (**c**) Iron reducing power.

The scavenging ability of AOE on DPPH free radicals is shown in Figure 2a. In the range of 0.5~10 mg/mL, the scavenging rate of Vc increased from 79.31% of 0.5 mg/mL to 94.25% of 6 mg/mL, then remained unchanged. The AOE clearance rate increased with the increase in concentration, reaching a maximum of 87.35% at a concentration of 10 mg/mL. The lower the IC_50_ value was, the higher clearance rate was. According to the 95% confidence interval, the IC_50_ of AOE and Vc were 3.126 mg/mL and 0.172 mg/mL.

#### 3.2.2. Hydroxyl Radical (·OH) Scavenging Ability of AOE

The hydroxyl radical scavenging ability of AOE is shown in Figure 2b. The hydroxyl radical scavenging rate of Vc basically remained unchanged within the range of 0.5~10 mg/mL. The AOE clearance rate increased with the increase in concentration, reaching a maximum of 65.79% when the concentration was 10 mg/mL. According to the 95% confidence interval, the IC_50_ values of AOE and Vc were 6.139 mg/mL and 0.006 mg/mL, respectively.

#### 3.2.3. Iron Ion Reduction Power of AOE

The determination of the reducing power of iron ions by AOE is shown in Figure 2c. In the range of 0.5~10 mg/mL, the absorption value of Vc increased with the increase in the concentration, and the absorption value of the AOE rose rapidly to less than 4 mg/mL and then tended to be flat. When the concentration was 10 mg/mL, both Vc and AOE reached the maximum absorption values, which were 235.50% and 196.20%, respectively.

### 3.3. AOE Alleviates Pathological Signs of DSS-Induced Colitis and Colonic Inflammation

**Figure 3:**
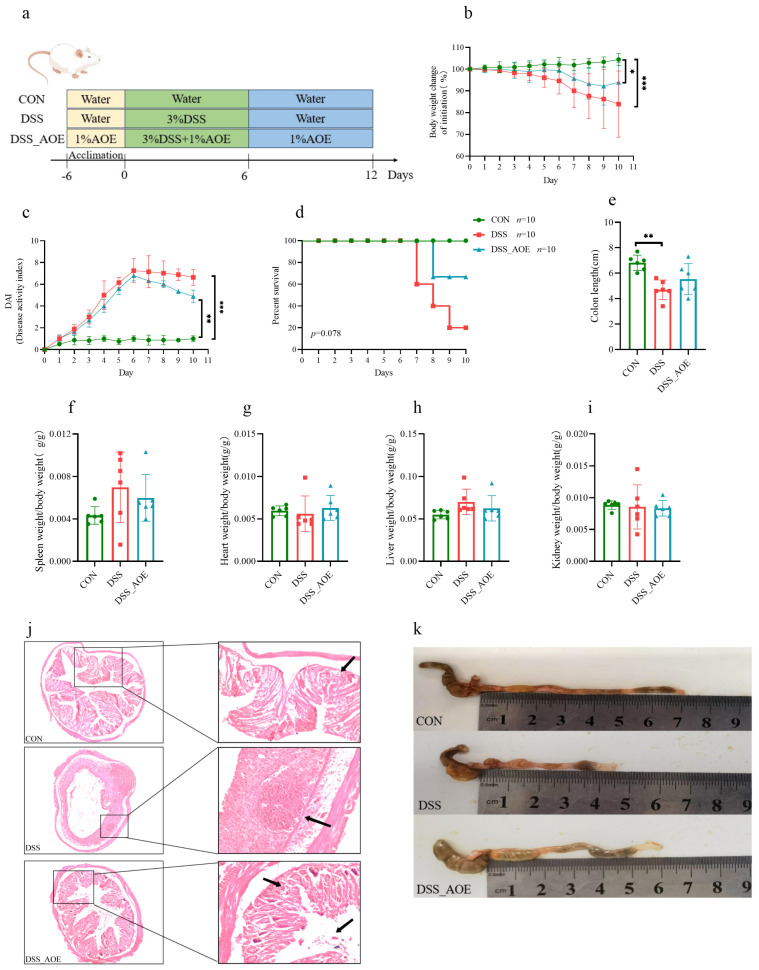
AOE alleviates colitis induced by DSS in mice. (**a**) The animal experimental protocol of this study. (**b**) Weight loss. (**c**) DAI score. (**d**) Percent survival. (**e**) Colon length. (**f**) Spleen index. (**g**) Liver index. (**h**) Heart index. (**i**) Kidney index. (**j**) H&E staining of colon. (**k**) The representative colon photos of mice in different groups. Note: * indicates *p* < 0.05, ** indicates *p* < 0.01, and *** indicates *p* < 0.001.

Colitis in mice that has been provoked by DSS serves as an effective model to mimic the typical clinical phenotype of inflammatory bowel disease (IBD) [30], exhibiting symptoms such as weight loss, bloody stool, and diarrhea. The administration of DSS led to a marked elevation in DAI scores, reflecting weight loss, fecal consistency, and fecal bleeding, thus confirming the completed elicitation of colitis in these mice. Notably, supplementation with AOE effectively alleviated these pathological features as demonstrated in Figure 3.

DSS treatment caused a decrease in body weight in mice (Figure 3b) and an elevation in the DAI score (Figure 3c). However, AOE administration was able to mitigate the weight loss to a certain extent and exert an inhibitory effect on the DAI score. Notably, throughout the experimental period, mice treated with AOE exhibited higher survival rates compared to the DSS group (Figure 3d). The impact of DSS on the visceral index is presented in Figure 3f–i. Colitis in mice that was provoked by DSS exhibited a shortened colon and enlarged spleen (Figure 3e,f,k), indicative of colon and systemic inflammation. Fortunately, AOE treatment restored colon length as anticipated. Furthermore, histopathological examination revealed extensive mucosal ulceration, infiltration of inflammatory cells, damage to the crypts, and disruption of epithelial cells in colitis mice (Figure 3j). However, mice treated with AOE displayed significant improvements in these symptoms. Additionally, AOE did not affect blood cells count in mice. Please refer to Table A3 for details.

### 3.4. Effects of AOE on Intestinal Immune, Antioxidant, and Barrier Functions of Colitis Induced by DSS

**Figure 4:**
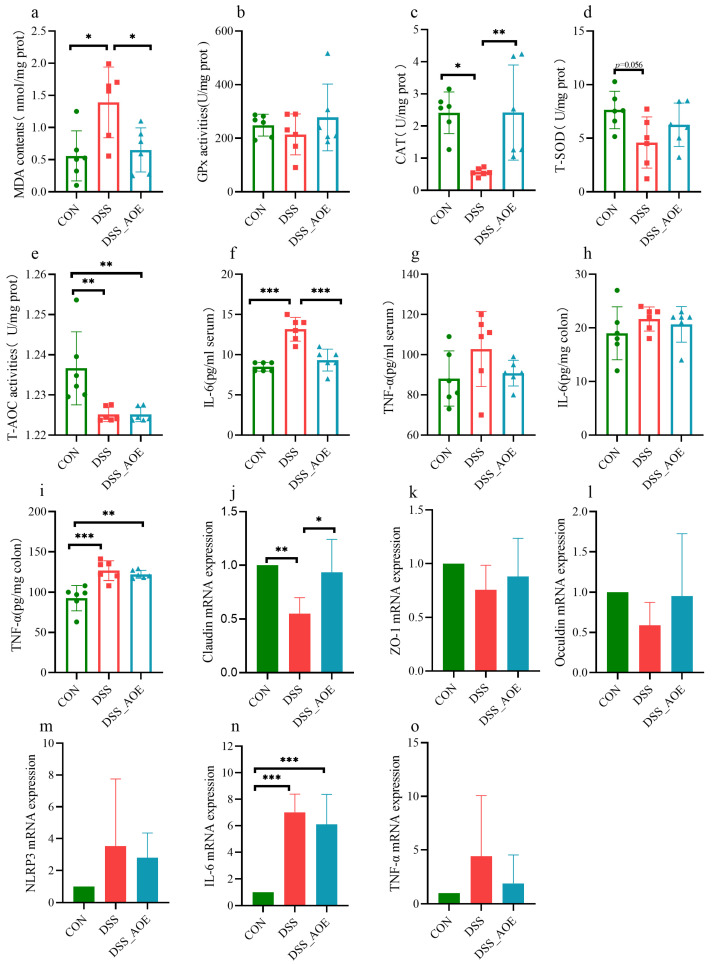
Changes in cytokines after AOE treatment. (**a**) MDA concentration in colon. (**b**) The activity of GPx in colon. (**c**) The activity of CAT in colon. (**d**) The activity of T-SOD in colon. (**e**) The activity of T-AOC in colon. (**f**) Serum IL-6 concentration. (**g**) Serum TNF-α concentration. (**h**) The concentration of IL-6 in colon. (**i**) The concentration of TNF-α in colon. (**j**) The relative expression of *Clauldin* mRNA in colon. (**k**) The expression level of *ZO-1* mRNA in colon. (**l**) The expression of *Occuldin* mRNA in colon. (**m**) The expression of *NLRP3* mRNA in colon. (**n**) The expression of *IL-6* mRNA in colon. (**o**) The expression of *TNF-α* mRNA in colon. Note: * indicates *p* < 0.05, ** indicates *p* < 0.01, and *** indicates *p* < 0.001.

Compared with the CON group, the DSS challenge resulted in an elevation of the colonic MDA content within colon tissue whereas AOE administration led to a reduction in MDA levels (Figure 4a). Additionally, the lower CAT levels observed in the colon tissue of DSS-treated mice were improved through the application of AOE (Figure 4c). The concentrations of IL-6 and TNF-α in both colon tissue and serum were measured using the ELISA method. Notably, colitis mice exhibited increased serum IL-6 and colonic TNF-α concentrations in contrast with the CON group (Figure 4f–i). However, following AOE treatment, the serum IL-6 level was reduced, indicating that AOE effectively inhibited the further progression of intestinal inflammation. To further assess the protective role of AOE in DSS-induced colitis, subsequent analysis was conducted to determine the mRNA expression levels of *NLRP3*, *IL-6*, and *TNF-α* in colon tissues (Figure 4m–o). The findings indicated that DSS administration resulted in an elevation of the relative expression of *IL-6* mRNA compared to the CON group. During treatment, the assessment of the intestinal epithelium’s integrity primarily entailed examining the colonic expression of tight junction proteins including *ZO-1*, *Occuldin*, and *Claudin* (Figure 4j–l). Interestingly, the addition of AOE to colitis mice upregulated the mRNA expression levels of *Claudin* compared to colitis mice without AOE treatment.

### 3.5. Effect of AOE on Gut Microbiota of DSS-Induced Colitis in Mice

**Figure 5:**
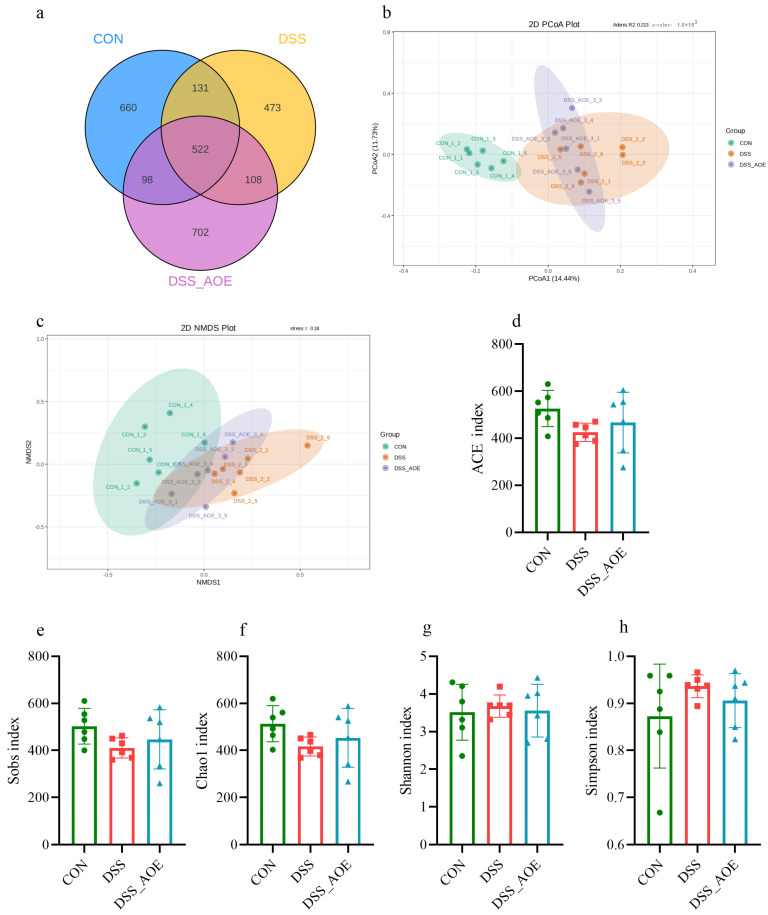
AOE ameliorates cecal microbiota of DSS-induced colitis mice. (**a**) Venn diagram. (**b**,**c**) Analysis of Beta diversity of the cecal microbiota by PCOA and NMDS index (**d**) ACE index. (**e**) Sobs index. (**f**) Chao1 index. (**g**) Shannon index. (**h**) Simpson index.

Extensive research has established a strong link between intestinal microbial imbalance and the inflammation of the intestinal mucosa. To further explore the consequence of AOE on the intestinal flora composition of DSS-treated mice, we conducted a comprehensive analysis using 16sRNA gene sequencing. The interaction between different groups of OUTs is clearly visualized in the Venn diagram (Figure 5a). The results indicate that the CON, DSS, and DSS_AOE groups harbored 1411, 1234, and 1430 OUTs, respectively, with shared percentages of 37.0%, 42.3%, and 36.5% across the groups. A Beta diversity assessment was conducted using principal coordinate analysis (PCoA), revealing that DSS treatment caused a significant shift in the intestinal flora away from the CON group. Interestingly, the DSS group and DSS_AOE group displayed a closer similarity in their microbial compositions (Figure 5b,c). Additionally, an Alpha diversity analysis was conducted to assess bacterial community diversity and richness. Notably, AOE treatment did not exert a significant effect on these metrics (Figure 5d–h).

**Figure 6:**
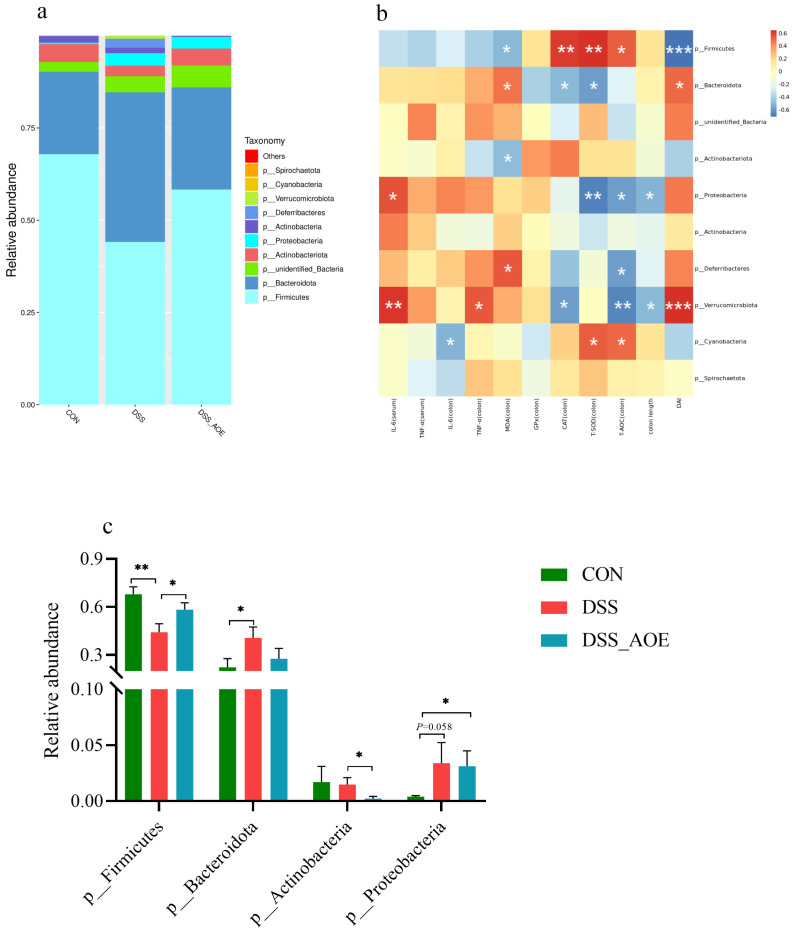
The effect of AOE on cecal microbiota at the phylum level in DSS-induced colitis mice. (**a**) Top-10-phylum-evel microbial contents. (**b**) Spearman correlation between top-10-phylum-level microbes and antioxidative parameters, inflammatory parameters, colon length, and DAI. (**c**) Phylum level microbial relative abundance. Note: * indicates *p* < 0.05, ** indicates *p* < 0.01, and *** indicates *p* < 0.001.

To achieve a more comprehension of gut microbiota composition and its association with gut diseases, we further analyzed the data at the phylum and genus levels. At the phylum level, the intestinal microflora in these mice was predominantly composed of *Firmicutes*, *Bacteroidota*, *unidentified_Bacteria*, *Actinobacteriota*, and *Proteobacteria* (Figure 6a). DSS treatment led to a significant decrease in the levels of *Firmicutes* compared to the CON group. However, the AOE treatment significantly increased the decrease in DSS-induced *Firmicutes*. It is worth noting that AOE also reduced the content of *Actinobacteria* compared to the DSS group (Figure 6c). Further, Spearman correlation analysis was conducted to investigate the relationship between the top-10-phylum-level microbes and antioxidative parameters, inflammatory parameters, colon length, and DAI. Correlation analysis revealed that *Firmicutes* exhibited a positive correlation with the CAT, T-SOD, and T-AOC levels in the colon but had a strong negative correlation with DAI (Figure 6b).

**Figure 7:**
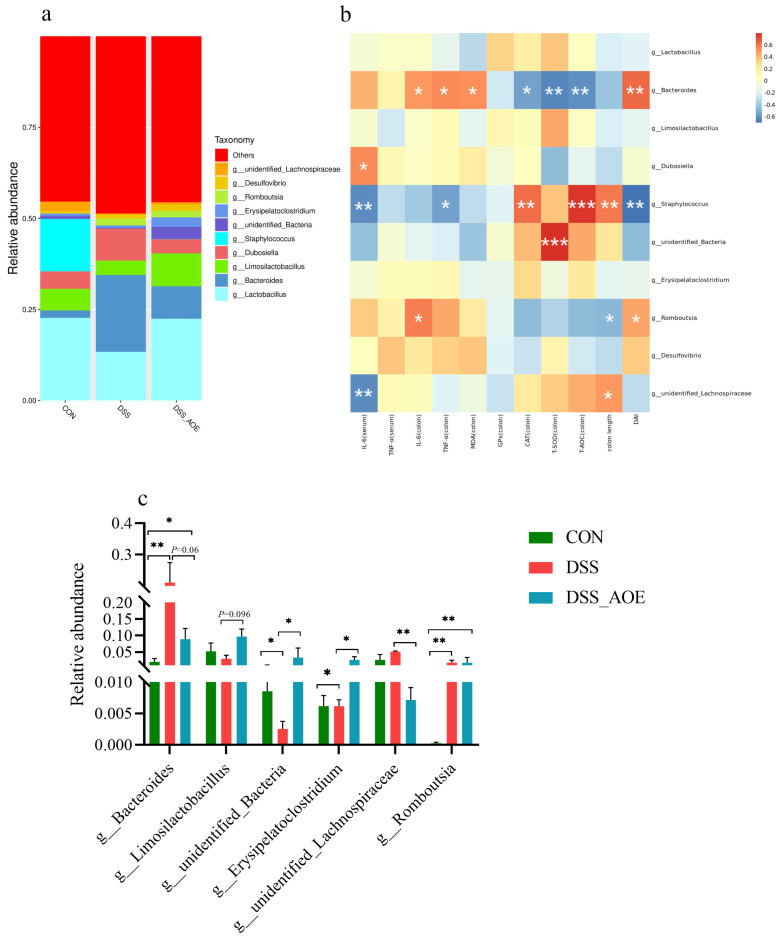
The effect of AOE on cecal microbiota at the genus level in DSS-induced colitis mice. (**a**) Top-10-genus-level microbial contents. (**b**) Spearman correlation between top-10-genus-level microbes and antioxidative parameters, inflammatory parameters, colon length, and DAI. (**c**) Genus-level microbial relative abundance. Note: * indicates *p* < 0.05, ** indicates *p* < 0.01, and *** indicates *p* < 0.001.

At the level of the genus, the fecal microflora was primarily dominated by *Lactobacillus*, *Bacteroides*, *Limosilactobacillus*, and *Dubosiella* (Figure 7a). Further analysis revealed that AOE significantly increased the relative abundance of *unidentified_Bacteria* and *Erysipelatoclostridium* in DSS-treated mice and showed a tendency to decrease *Limosilactobacillus* while decreasing the relative richness of *Bacteroides* and *unidentified_Lachnospiraceae* (Figure 7c). Spearman correlation analysis revealed that Bacteroides exhibited a positive correlation with the colon IL-6, TNF-α, MDA, and DAI while having a negative correlation with CAT, T-SOD, and T-AOC. On the contrary, Staphylococcus had a positive correlation with the colon CAT, T-AOC, and colon length while having a negative correlation with serum IL-6, colon TNF-α, and DAI (Figure 7b). These findings provide valuable insights into the complex interactions between AOE, intestinal flora, and intestinal mucosal inflammation.

### 3.6. Effect of AOE on Microbial BA Metabolism of DSS-Induced Colitis in Mice

**Figure 8:**
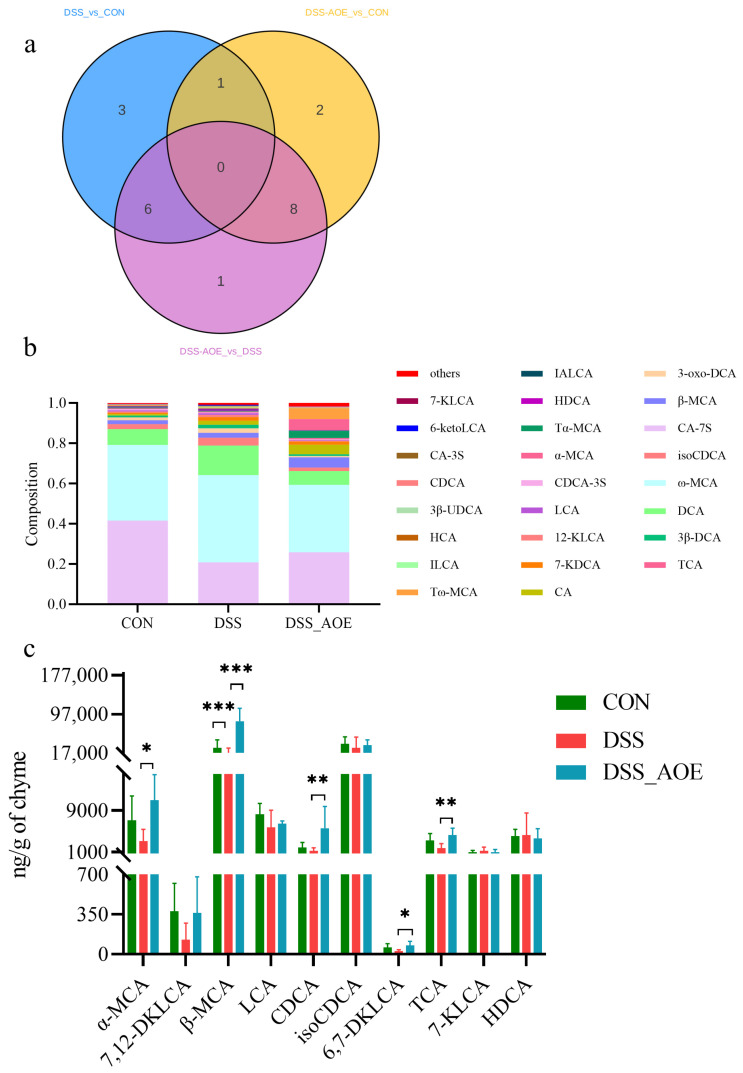
Bile acid correlation analysis of cecum. (**a**) Differential bile acid metabolites. (**b**) Top 25 BAs. (**c**) BA abundance maps from different groups. Note: * indicates *p* < 0.05, ** indicates *p* < 0.01, and *** indicates *p* < 0.001.

BAs exert a profound influence on the composition and abundance of intestinal flora. Their direct killing effect helps reduce the number of harmful bacteria while their endocrine function prompts other cells in the intestinal cavity to secrete antibacterial substances, thereby enhancing the mucosal immunity of the host [31]. In this study, we employed LC/QTRAP-MS for a comprehensive metabolomic analysis of cecal-content BAs. The Venn diagram of differentially abundant bile acid metabolites among different groups is shown in Figure 8a. Our findings revealed the presence of 39 commonly occurring BAs across all samples, with the top 25 BAs depicted in Figure 8b. Notably, AOE treatment significantly elevated the levels of α-MCA, β-MCA, CDCA, 6,7-DKLCA, and TCA in colitis mice, as shown in Figure 8c.

**Figure 9:**
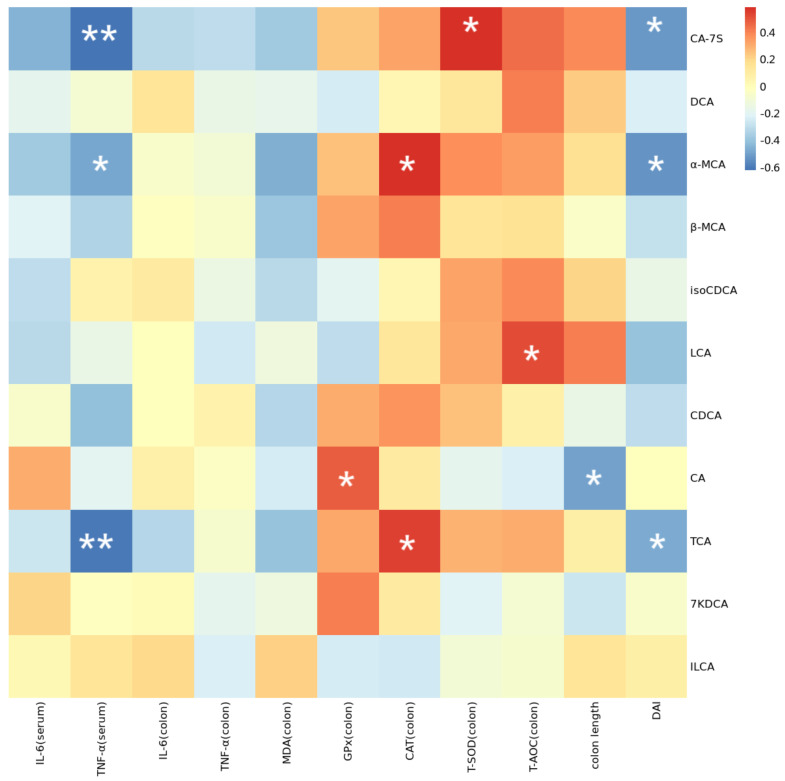
Spearman correlation between BAs and antioxidative parameters, inflammatory parameters, colon length, and DAI. Note: * indicates *p* < 0.05, ** indicates *p* < 0.01.

Further, Spearman correlation analysis was conducted to investigate the relationship between BAs and antioxidative parameters, inflammatory parameters, colon length, and DAI. Correlation analysis revealed that CA-7s displayed a positive correlation with colon T-SOD and exhibited a negative correlation with DAI. The levels of α-MCA and TCA were negatively correlated with serum TNF-α and DAI and positively correlated with CAT (Figure 9).

**Figure 10:**
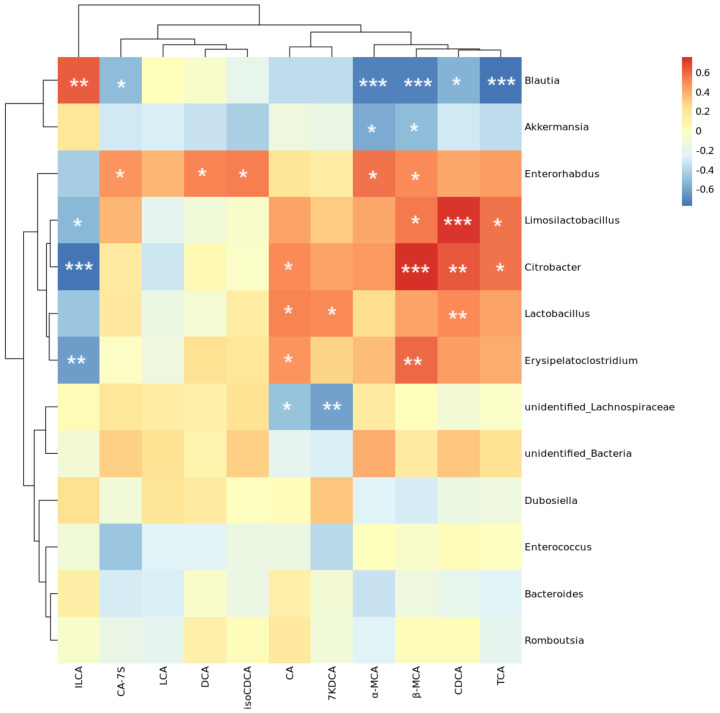
Spearman correlation between cecal microbiota and BAs. Note: * indicates *p* < 0.05, ** indicates *p* < 0.01, and *** indicates *p* < 0.001.

To further investigate the intricate relationship between fecal bile acid levels and intestinal flora imbalance during colitis, we conducted a Spearman correlation analysis. The results revealed intriguing relationships: CDCA levels positively correlated with *Lactobacillus*, *Citrobacter*, and *Limosilactobacillus* abundances while exhibiting a negative correlation with *Blautia* abundance. β-MCA levels showed positive correlations with *Erysipelatoclostridium*, *Citrobacter*, *Limosilactobacillus*, and *Enterorhabdus* abundances and negative correlations with *Akkermansia* and *Blautia* abundances. Similarly, α-MCA levels positively correlated with *Enterorhabdus* abundance and negatively with *Akkermansia* and *Blautia* abundances (Figure 10). These findings suggest that changes in fecal bile acid profiles during colitis are closely linked to alterations in the intestinal microbiota.

## 4. Discussion

*Artemisia ordosica Krasch*, a distinguished herb from the *Artemisia* genus of the *Asteraceae* family, is renowned for its medicinal value. Due to their rich nutritional content and bioactive compounds, various species of *Artemisia*, along with their extracts, are extensively used in traditional healing practices around the world. In traditional Chinese medicine, herbs are typically prepared using the decoction method [32]. This technique effectively breaks down the plant’s cell walls, facilitating the extraction of valuable bioactive compounds from within the cells. Former research endeavors have shown that *Artemisia* plants exhibit antioxidant effects. Both the ether extract of *Artemisia ordosica Krasch* and the ethanol extract of *Artemisia argyi* have shown strong antioxidant capacity [22,33]. Flavonoids and phenols can act as suppliers of electrons or hydrogen, mitigating free radical generation and eliminating free radicals [34,35]. In this study, we found that AOE exhibited strong antioxidant activity in vitro. Additionally, LC-MS/MS analysis revealed that flavonoids and phenolic compounds were the most abundant components in AOE. Therefore, we hypothesize that flavonoids and phenolic compounds are the effective bioactive constituents responsible for the antioxidant activity of AOE.

IBD, a health-threatening disease, is experiencing a rapid surge worldwide. Numerous epidemiological and experimental studies have established that dietary intervention offers a viable approach to prevent ulcerative colitis [36,37]. Consequently, developing effective dietary products aimed at colitis prevention remains a prime concern. DSS-induced colitis primarily results in intestinal ailments such as pathological damage, shortened colon lengths, and abnormal stool consistency [38]. Our study revealed that AOE reversed symptoms like weight loss, DAI elevation, colon shortening, and pathological damage in colitis mice, indicating its potential in mitigating colitis-induced bodily harm. One of the primary mechanisms underlying IBD pathogenesis is the overproduction of reactive oxygen species (ROS), which leads to oxidative stress and further exacerbates intestinal inflammation. Our findings demonstrate that AOE supplementation enhances the antioxidant capacity in DSS mice and reduces MDA levels, thereby mitigating excessive ROS generation and bolstering antioxidant enzyme activity in animals. This highlights the potential of AOE to mitigate oxidative stress and inflammation, crucial factors in IBD progression. Furthermore, UC patients often exhibit symptoms beyond intestinal diseases, affecting other organs as well [39]. The spleen, a crucial immune organ, may become enlarged in colitis patients [40]. Our study showed that AOE treatment reversed the spleen index, further suggesting its effectiveness in mitigating colitis-related complications and enhancing systemic health. A critical feature of IBD is the disruption of the intestinal barrier, which allows harmful substances to translocate into the bloodstream, triggering systemic inflammation. IL-6 and TNF-α are pro-inflammatory cytokines while tight junction proteins play a vital role in preventing harmful substances from entering the bloodstream through the intestinal cavity [41]. Our study demonstrated that AOE supplementation upregulated the expression of colon tight-junction-related proteins while reducing serum levels of pro-inflammatory cytokines like IL-6 and TNF-α. This suggests that AOE can help restore intestinal barrier integrity and reduce systemic inflammation in colitis.

The gut microbiota, consisting of trillions of microorganisms, plays a crucial role in maintaining intestinal homeostasis, influencing various physiological processes including BA metabolism [42]. The gut microbiota is central to the biotransformation of primary BAs into secondary BAs, a process that directly affects the functionality of bile acid signaling pathways. In particular, the gut microbiota is capable of deconjugating and transforming conjugated primary bile acids into secondary bile acids via enzymatic activities such as 7α-dehydroxylation [43]. This microbial-mediated conversion is essential for maintaining BA homeostasis and, by extension, gut health.

In patients with IBD and in experimental animal models, the dysregulation of the gut microbiota composition is commonly observed. Notably, genera such as *Lactobacillus*, *Bacteroides*, *Romboutsia*, and *Limosilactobacillus* are frequently implicated in this dysbiosis [44,45]. Our findings indicate that AOE treatment effectively rejuvenates the abundance of these key bacterial genera in rats with DSS-induced colitis. Particularly, *Limosilactobacillus*, a genus involved in bile acid metabolism, has been shown to help maintain intestinal homeostasis by enhancing the production of short-chain fatty acids and suppressing inflammatory pathways such as the Fas/Fasl signaling axis and the TLR4/NF-κB pathway [46]. Importantly, we observe that AOE treatment increases the relative abundance of *Limosilactobacillus*, which may contribute to its beneficial effects in promoting intestinal health.

BAs are not only essential for digestion and lipid absorption but also possess anti-inflammatory properties and influence lipid metabolism, glucose regulation, and the energy balance [12]. Among the bile acids, CDCA, LCA, DCA, and CA are the primary endogenous ligands of the farnesoid X receptor (FXR), with CDCA showing the most potent activation of FXR, followed by DCA, LCA, and CA [47,48]. The activation of the FXR by these bile acids modulates several critical processes including the regulation of intestinal barrier integrity and the suppression of inflammation. Reduced levels of unconjugated and secondary bile acids, such as CDCA and LCA, have been associated with impaired FXR activation, which can compromise anti-inflammatory pathways and contribute to IBD pathogenesis [49,50]. Additionally, supplementing with LCA and CDCA has been shown to improve DSS-induced barrier dysfunction and colonic inflammation [51]. The administration of CDCA to piglets has been found to bolster intestinal barrier function by suppressing the expression of TNF-α and IL-6 [52]. In this context, our findings align with previous research, suggesting that AOE treatment elevates CDCA levels in vivo, potentially through microbiota-mediated bile acid conversion. This elevation in CDCA may enhance FXR activation, strengthen intestinal barrier integrity, and mitigate inflammatory responses in DSS-induced colitis models.

Our study also explored the intricate relationship between intestinal microbiota composition and bile acid metabolism. Notably, *Lactobacillus* species play a pivotal role in converting primary BAs into secondary BAs, such as DCA and LCA, through 7α-dehydroxylation. Previous research has demonstrated that Lactobacillus can alleviate colitis by promoting mucus production, supporting intestinal epithelial regeneration, and dampening inflammatory responses [53,54,55]. Importantly, we found a positive correlation between *Lactobacillus* abundance and CDCA levels in AOE-treated DSS mice. This suggests that AOE may indirectly promote the growth of *Lactobacillus*, thereby enhancing secondary BA production and contributing to the restoration of intestinal homeostasis. By elevating CDCA levels, AOE could enhance FXR signaling, which further supports gut barrier function and reduces inflammation in colitis models.

In addition to *Lactobacillus*, we observed that AOE administration also significantly increased the levels of α-MCA and β-MCA, two metabolites derived from primary BAs such as CDCA. Interestingly, we found a negative correlation between these secondary BAs and the abundance of *Akkermansia*, a microbiota genus associated with the stimulation of pro-inflammatory cytokines like IL-6 [56]. This observation suggests that AOE may regulate the levels of α-MCA and β-MCA to suppress *Akkermansia* proliferation, thereby reducing the secretion of inflammatory cytokines such as IL-6. By modulating both the microbiota composition and bile acid metabolism, AOE appears to exert a dual beneficial effect, mitigating DSS-induced inflammation and reinforcing intestinal barrier function.

In summary, our study provided strong evidence that AOE not only exhibits potent antioxidant activity in vitro but also alleviates DSS-induced colitis through a mechanism that combines the regulation of gut microbiota and bile acid metabolism. By promoting the growth of beneficial microbiota, AOE enhances the conversion of primary BAs into secondary BAs, which in turn activates FXR signaling to strengthen intestinal barrier integrity and reduce inflammation. Additionally, the regulation of secondary BAs, including α-MCA and β-MCA, appears to suppress the pro-inflammatory activity of *Akkermansia*, further contributing to the anti-inflammatory effects of AOE. These findings underscore the importance of microbiota–bile acid interactions in the pathophysiology of IBD and highlight AOE as a potential therapeutic strategy for modulating the gut microbiota and its associated bile acid metabolism to restore intestinal homeostasis and reduce inflammation.

## 5. Conclusions

In conclusion, the results of our study suggest that feeding AOE may prevent DSS-induced colitis by regulating the inflammatory response, restoring the gut barrier and microbiota, and regulating intestinal bile acid metabolism. These findings provide preliminary experimental data for the potential use of AOE in intestinal diseases and reveal a new role for AOE in preventing intestinal dysfunction. Furthermore, our findings only show that AOE prevents DSS-induced colitis in a preventive manner and need to be validated in a therapeutic setting.

## Data Availability

Data are contained within the article.

## Appendix A

**Table A1:** Disease activity index, DAI.

Score	Weight Loss (%)	Stool Consistency	Fecal Blood Content
0	None	Normal	None
1	1–5%	-	
2	6–10%	Loose stool	Occult blood
3	11–15%	-	
4	>16%	Diarrhea	Hemorrhage/Gross bleeding

**Table A2:** Primer sequences used for the study.

Primer Name	Reference Sequence	Primer Sequence (5′-3′)
IL-6	NM_001314054.1	F: CTGCAAGAGACTTCCATCCAG
		R: AGTGGTATAGACAGGTCTGTTGG
TNF-α	NM_007021.4	F: TGAGGTCAATCTGCCCAAGT
		R: GGGGTCAGAGTAAAGGGGTC
NLRP3	NM_001359638.1	F: ATTACCCGCCCGAGAAAGG
		R: TCGCAGCAAAGATCCACACAG
ZO-1	XM_054378715.1	F: GAGCAGGCTTTGGAGGAGAC
		R: TGGGACAAAAGTCCGGGAAG
Occludin	XM_054351382.1	F: TTGAAAGTCCACCTCCTTACAGA
		R: CCGGATAAAAAGAGTACGCTGG
Claudin	NM_021101.5	F: TGCCCCAGTGGAAGATTTACT
		R: CTTTGCGAAACGCAGGACAT
GAPDH	NM_001357943.2	F: ATGGGAAGCTTGTCATCAACG
		R: AAGACACCAGTAGACTCCACG

**Table A3:** Blood cell analysis ^1^.

Item (Unit)	CON	DSS	DSS_AOE	*p*-Value
WBC (×10^9^/L)	2.06 ± 0.83	2.44 ± 0.57	1.92 ± 0.54	0.36
LYM (×10^9^/L)	1.26 ± 0.65	1.30 ± 0.38	1.06 ± 0.41	0.69
MID (×10^9^/L)	0.27 ± 0.12	0.26 ± 0.15	0.20 ± 0.11	0.59
GRAN (×10^9^/L)	0.53 ± 0.12 ^b^	0.88 ± 0.13 ^a^	0.70 ± 0.23 ^ab^	0.01
RBC (×10^12^/L)	5.09 ± 0.45	4.47 ± 0.59	4.49 ± 0.45	0.08
HGB (g/L)	79.33 ± 9.65	71.00 ± 11.36	70.50 ± 8.77	0.30
PCV (%)	30.32 ± 2.87	26.92 ± 3.21	27.40 ± 2.76	0.13
MCV (fL)	59.63 ± 1.01	60.36 ± 1.94	61.16 ± 3.10	0.51
MCH (pg)	15.53 ± 0.58	15.76 ± 0.71	15.62 ± 0.93	0.89
MCHC (g/L)	260.83 ± 8.28	262.20 ± 11.91	256.6 ± 12.84	0.51
RDW-SD (%)	23.59 ± 3.18	27.10 ± 2.50	27.11 ± 4.97	0.20
RDW-CV (%)	13.9 ± 0.60	15.20 ± 1.09	15.00 ± 2.11	0.24
PLT (×10^9^/L)	334.70 ± 123.50 ^b^	518.00 ± 130.16 ^a^	396.60 ± 74.83 ^ab^	0.04
MPV (fL)	6.87 ± 0.46	6.86 ± 0.33	7.19 ± 0.70	0.49
PDW (%)	8.58 ± 1.07	7.56 ± 1.53	8.29 ± 1.64	0.47
PCT (%)	0.23 ± 0.08	0.35 ± 0.20	0.29 ± 0.19	0.46
P-LCR (%)	4.51 ± 2.03	3.20 ± 1.74	3.97 ± 2.16	0.51

^1^ Abbreviations: WBC: white blood cell, LYM: lymphocyte, MID: intermediate cell, GRAN: granulocyte, RBC: red blood cell, HGB: hemoglobin, PCV: packed cell volume, MCV: mean corpuscular volume, MCH: mean corpuscular hemoglobin, MCHC: mean corpuscular hemoglobin concentration, RDW-SD: red blood cell volume distribution width-SD, RDW-CV: red blood cell volume distribution width-CV, PLT: platelet, MPV: mean platelet volume, PDW: platelet distribution width, PCT: thrombocytocrit, and P-LCR: platelet larger cell ratio. Note: Different lowercase letters indicate significant differences while the absence of letters indicates no significant difference.
